# Fine-tuning biexcitons-plasmon coherent states in a single nanocavity

**DOI:** 10.1515/nanoph-2023-0304

**Published:** 2023-07-25

**Authors:** Kun Liang, Lei Jin, Xuyan Deng, Ping Jiang, Li Yu

**Affiliations:** School of Electronic Engineering, Beijing University of Posts and Telecommunications, Beijing, China; School of Science, Beijing University of Posts and Telecommunications, Beijing, China; School of Science Microelectronics and Data Science, Anhui University of Technology, Maanshan, China; State Key Laboratory of Information Photonics and Optical Communications, School of Science, Beijing University of Posts and Telecommunications, Beijing, China

**Keywords:** biexcitons-plasmon coupling, double Rabi splitting, dynamic control, nano-optics, two-dimensional material

## Abstract

A tunable plexcitonic material that sustains multimode hybridization is highly desirable, which is vital for advanced quantum devices. However, the research about regulations of biexcitons-plasmon coherent states has rarely been reported. Here we apply single-nanoparticle scattering spectroscopy correlative with SEM imaging to identify biexcitons-plasmon interaction in a metal-semiconductor hybrid structure composed of a single Au@Ag nanoparticle, J-aggregates molecules and tungsten disulfide (WS_2_) monolayer. The mode competition within the localized plasmonic hotspots (∼240 nm^3^) is revealed by continuously regulating the J-aggregates spacer. Two distinct anticrossings are observed at both excitons resonances, and large double Rabi splittings (137 meV and 124 meV) are obtained successfully. We establish experimentally that J-aggregates and WS_2_ monolayer are responsible for the middle polariton states, while plasmon rarely contributes. Further calculations show that plasmonic nanocavity enables coherent energy exchange with different excitons by providing a highly enhanced localized E-field. In addition, we find that the multimode coupling strengths can be efficiently tuned by changing the cavity morphology and environment temperature, where the tuning spectral accuracy can reach up to 1 nm. Our findings uncover the distinctive properties of biexcitons-plasmon polaritons, suggest an easily obtainable multiqubit states platform, and open up a new way to construct nanoscale photonic devices.

## 1 Introduction

Room-temperature strong coupling between quantum emitters (QEs) and plasmon polaritons has raised much attention in the optics and quantum physics communities, since it reveals a plethora of intriguing phenomena such as vacuum Rabi splitting [1, 2], Bose–Einstein condensation [3, 4], optical Stark effect [5, 6], entanglement [7, 8], and quantum network [9, 10]. It differs from weak coupling [11], where only the spontaneous emission rate was modified. Strong coupling [12] generates mixed states which possess both photonic and excitonic characters. The coherent energy transfer rate in the strong coupling regime surpasses the dissipation and decoherence rates. With the flourishing of nanofabrication technology and quantum electrodynamics, strong coupling systems exhibit great potential in many powerful applications, such as quantum light source [13, 14], ultrafast optical switching [15], superfluidity [16], single-molecule sensing [17], and quantum computing [18]. Up to now, a variety of plasmonic nanostructures have been proposed to minimize the mode volumes *V*_eff_ since the coupling strength g is inversely proportional to $\sqrt{V_{e\mathrm{ff}}}$ [19]. Recently, even ultrasmall *V*_eff_ below 1 nm^3^ has been realized in the nanoparticle-on-mirror system [20]. The plasmon-exciton strong coupled system has been successfully demonstrated at the single emitter level [21–24], building a firm foundation for functional quantum plasmonic devices. At the single nanocavity level, strong coupling between plasmon modes and 2D materials such as WS_2_ [25, 26] and WSe_2_ [27, 28] monolayer has been demonstrated, providing novel routes for active and plasmonics applications.

Despite these remarkable developments, the existing researches mainly focus on hybrid systems consisting of cavities and homogeneous quantum emitters, which only generate two coherent states. Considering the profound quantum effects and technological frontiers in the multi-mode coupled systems [29–35] (e.g., quantum network, quantum computing, and nanolasers), strong coupling among three excitations is highly desirable. However, few have been reported in the single QEs-plasmonic nanocavity system due to the large Ohmic loss of metal [36]. One major challenge has to be overcome to trigger biexciton-plasmon strong coupling: The local electric field at heterogeneous QEs locations must be sufficiently enhanced to simultaneously empower coherent energy transfer in two different plasmon-exciton coupling subsystems. Relevant experimental [37, 38] and theoretical work [39] emerged as the extension of two-mode strong coupling. Cuadra et al. have successfully demonstrated three intermixed plasmon–exciton–trion coherent states at the single nanoparticle level under cryogenic conditions, which consist of an individual silver nanoantenna and monolayer WS_2_ hybrid system [26]. Recently, Lan et al. realized active tuning of strong plasmon–exciton–trion coupling in Si/WS_2_/Au nanocavities by increasing the laser power [40]. In another work, Zhou et al. [41] demonstrated plasmon-assisted coherent energy transfer between far-detuned QEs, setting the foundation for future quantum networks.

Inspired by these pioneering works, in this study, we demonstrate strong interactions among plasmons in an individual Au@Ag nanocavity, Frenkel excitons in TDBC J-aggregates, and Wannier excitons in monolayer WS_2_. Three hybrid states formed by the biexcitons-plasmon coupling and double Rabi splitting phenomenon (137 meV and 124 meV) were observed under ambient conditions. Furthermore, we reveal the mode competition between two coupled excitonic modes and optimize the multimode coupling nanosystems by controlling the coated J-aggregates spacers. Theoretical calculations indicate that the cavity morphology and environment temperature can tailor the degree of biexcitons-plasmon coupling. Modification in the dielectric constant of the WS_2_ monolayer induced by tuning the temperature has also been extracted, which can well explain the temperature-resolved scattering spectra.

## 2 Results and discussion

### 2.1 Introducing biexcitons-plasmon coupling in a Au@Ag/J-aggregates/WS_2_ nanocavity

**Figure 1:**
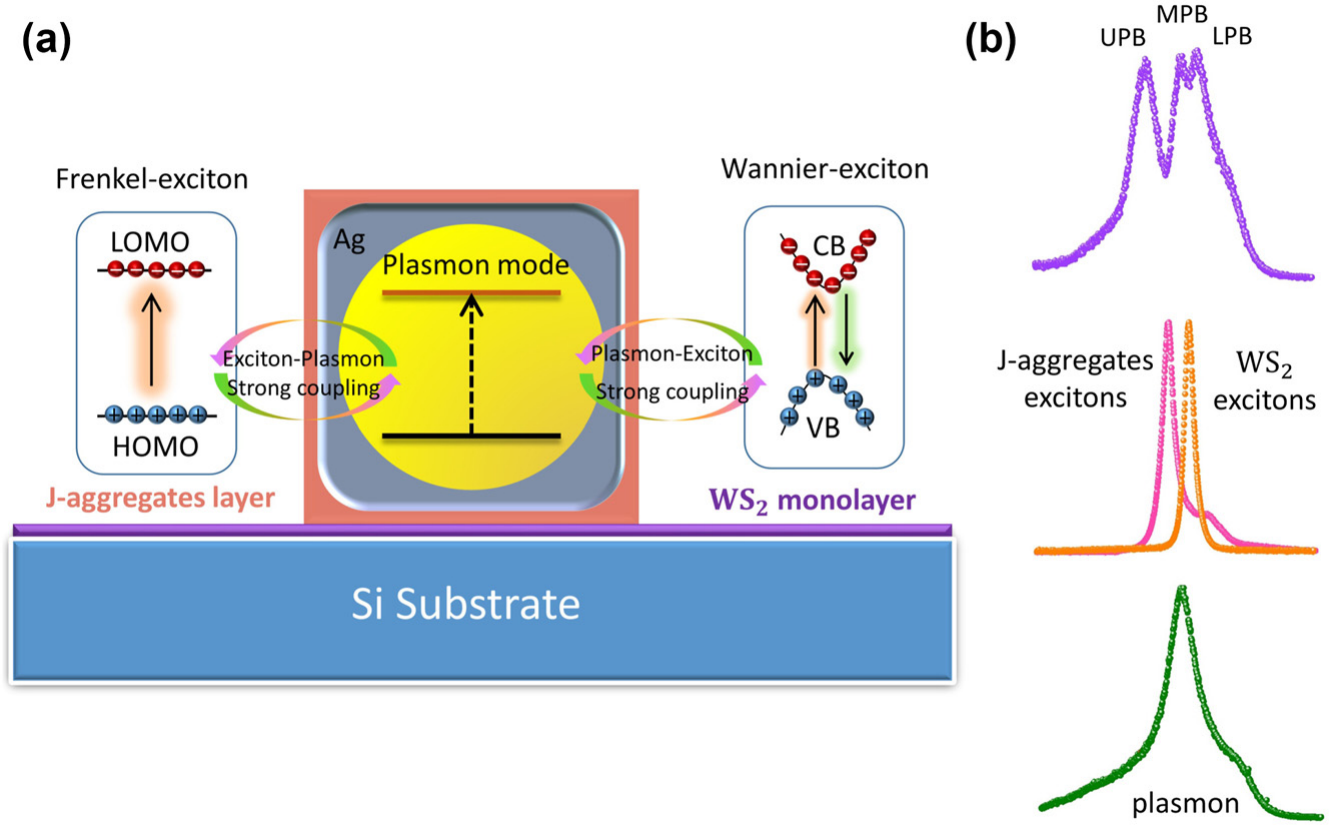
Concept of biexcitons-plasmon strong coupling between detuned excitonic materials and an individual metallic nanocavity. (a) Schematic showing a delicately designed QEs-nanoantenna structure which empowers simultaneous energy exchange with both Frenkel-excitons and Wannier-excitons. Ultimately, the newly formed biexcitons-plasmon hybrid states can possess the characteristics of both QEs. (b) Measured spectra of uncoupled components (lower) and multi-mode strong coupled nanosystem (upper) exhibit a distinct double Rabi-splitting signature.

As depicted in Figure 1a, the coupled system in this work is composed of Au@Ag core–shell nanocavity coating with TDBC J-aggregates, which is positioned on the surface of monolayer WS_2_. As an essential component, plasmonic nanocavity plays a critical role in providing a highly-enhanced local electric field, empowering different strong coupling channels with heterogeneous QEs. Frenkel-type excitons are formed in molecular materials, typically with small exciton radii and asymmetric charge distribution [42]. Therefore, they exhibit strong polar chemical properties. However, due to their formation inside molecules and weak interactions between electrons and holes, Frenkel-type excitons have short lifetimes, and it is challenging to achieve long-lifetime luminescence. In contrast, Wannier-type excitons formed in crystals typically have longer lifetimes and larger exciton radii, along with lower charge transfer efficiency [43]. Here we take advantage of multimode coupling among plasmon, Frenkel-type excitons, and Wannier-type excitons, which enable the possibility to exhibit the best features of different excitonic materials (see Figure 1a). The electromagnetic coupling between the localized plasmon resonance and J-aggregates/WS_2_ excitons is different due to their spatial distribution variations. The excitons in J-aggregates are of Frenkel type, which is characterized by its strong binding energy (order of 1 eV), large dipole moment (0.7 e nm), and small Bohr radius (1 nm) [44]. Ultrathin J-aggregates monolayer is used to realize single-exciton level strong coupling [19]. It is demonstrated that only the J-aggregate exciton within the sufficient localized E-field can be involved in the strong coupling process. In our system, strong electromagnetic coupling between J-aggregates excitons and plasmon modes mainly happens at the sharp corners of Au@Ag nanorods. On the other hand, excitons in WS_2_ are of Wannier type, which is delocalized over several unit cells and possess larger scattering cross sections. Monolayer WS_2_ is a direct bandgap semiconductor, leading to excitons with enormous binding energies (700 meV) [45]. Moreover, the optical property of single-crystalline WS_2_ is uniform across the entire two-dimensional flake, which is beneficial to form robust pl-exciton systems. To realize biexcitons-plasmon coupling, the spectra properties of the three uncoupled components are carefully investigated before sample assembling (as shown in Figure 1b). The PL spectra measured for the monolayer WS_2_ (under ambient condition) shows a resonance peak at *E*_*x*_ = 2029 meV, while the measured absorption resonance for the J-aggregates is *E*_*j*_ = 2099 meV. The dark-field scattering spectrum of a typical Au@Ag nanocavity shows a broad resonance (*E*_*pl*_ = 2075 meV) in the middle of the two excitonic materials. The decay rate of the WS_2_ excitons is *γ*_*x*_ = 26 meV (measured by absorption spectrum in Figure S4) or 19 meV (measured by photoluminescence spectrum in Figure 1b). It is noteworthy that the extracted decay rate of J-aggregates (*γ*_*j*_ = 25 meV) and WS_2_ is relatively small compared to the plasmon nanocavity (*γ*_*pl*_ = 144 meV). When two adjacent excitonic modes spectral overlap with the plasmonic nanocavity, it might be possible to form a multi-mode coupling system. The top of Figure 1b shows the scattering spectra (solid purple curves) measured for the WS_2_/J-aggregates/Au@Ag nanocavity irradiated using a halogen lamp. Three new energy branches will be formed if excitons of both the J-aggregates and WS_2_ interact intensively with the plasmonic mode, which is marked as the upper polariton branch (UPB), the middle polariton branch (MPB), and the lower polariton branch (LPB).

### 2.2 The properties of plasmon-exciton subsystems

**Figure 2:**
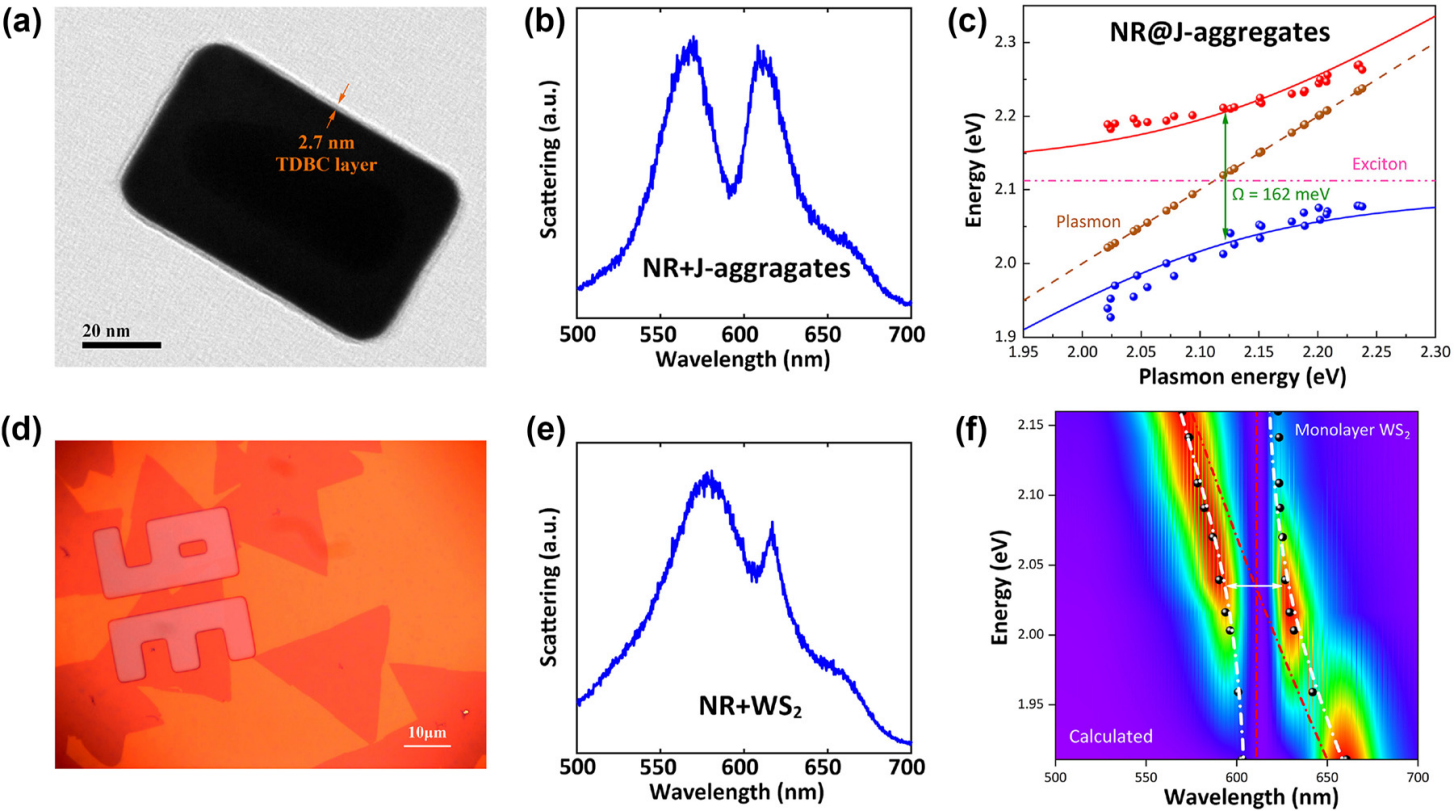
Single-mode plasmon-exciton strong coupling. (a) High-resolution TEM image of Au@Ag nanorod coated with 2.7 nm TDBC J-aggregates layers. (b) Scattering spectra of resonate Au@Ag nanorod and J-aggregates hybrid nanosystem. (c) Dispersion of plexciton with UPB and LPB varied as a function of plasmon energy, presenting a Rabi splitting up to 162 meV. (d) Bright-field image of WS_2_ monolayer collected by a 100× objective. (e) Scattering spectra of red-detuned Au@Ag nanorod and monolayer WS_2_ nanosystem. (f) Calculated dispersion of single Au@Ag nanocavity and WS_2_ strong coupled system, presenting a Rabi splitting of 110 meV.

Before diving into the complex coupling among nanocavity, J-aggregates and WS_2_, we first investigate the properties of the two plexcitonic sub-systems. By controlling the Ag shell’s thickness in the growth process, the localized surface plasmon resonance of Au@Ag nanocavity was carefully tuned from 651 nm to 565 nm, which crosses the exciton resonance of both J-aggregates and WS_2_. The fine-tuning originates from the high sensitivity of LSPR mode to the aspect ratio [19]. In Figure 2, the scattering spectrum of the nanocavity/J-aggregates hybrid shows two peaks and a dip at 592 nm, which is the exciton resonance of J-aggregates. To attain the dispersion curve, the scattering spectra of nanocavity/J-aggregates hybrid with different Ag shell thicknesses were measured. As shown in Figure 2c, the newly formed hybrid states’ eigenenergies were extracted, exhibiting a distinct anticrossing phenomenon. A large Rabi splitting (∼162 meV) was observed at resonant conditions. Compared to the plasmon and exciton linewidths (*γ*_*pl*_ = 150 meV, *γ*_*j*_ = 25 meV), the Rabi splitting energy Ω_*pj*_ > $\frac{\gamma_{pl}+\gamma_{j}}{2}$, which indicates the nanocavity/J-aggregates hybrid has entered the strong coupling regime. Next, we investigate the strong coupling between plasmon and the WS_2_ excitons. The WS_2_ monolayers synthesized by chemical vapor deposition were transferred to Si/SiO_2_ substrates with a 100 nm thick SiO_2_ film. In Figure 2d, the dark-field image of the wafer was demonstrated, showing a firm and direct contact between nanocavities and WS_2_ monolayers. Two new eigenstates were formed at the flanks of the WS_2_ excitonic resonance (*λ*_*x*_ = 612 nm), as shown in Figure 2e. In Figure 2f, the energy dispersion of the upper polariton branches (UPB) and the lower polariton branches (LPB) exhibit anticrossing behavior. A Rabi splitting of 110 meV is observed, which close to satisfy the criteria for strong coupling (Ω_*px*_ > $\frac{\gamma_{pl}+\gamma_{x}}{2}$).

### 2.3 Mode competition within the localized plasmonic hotspots

**Figure 3:**
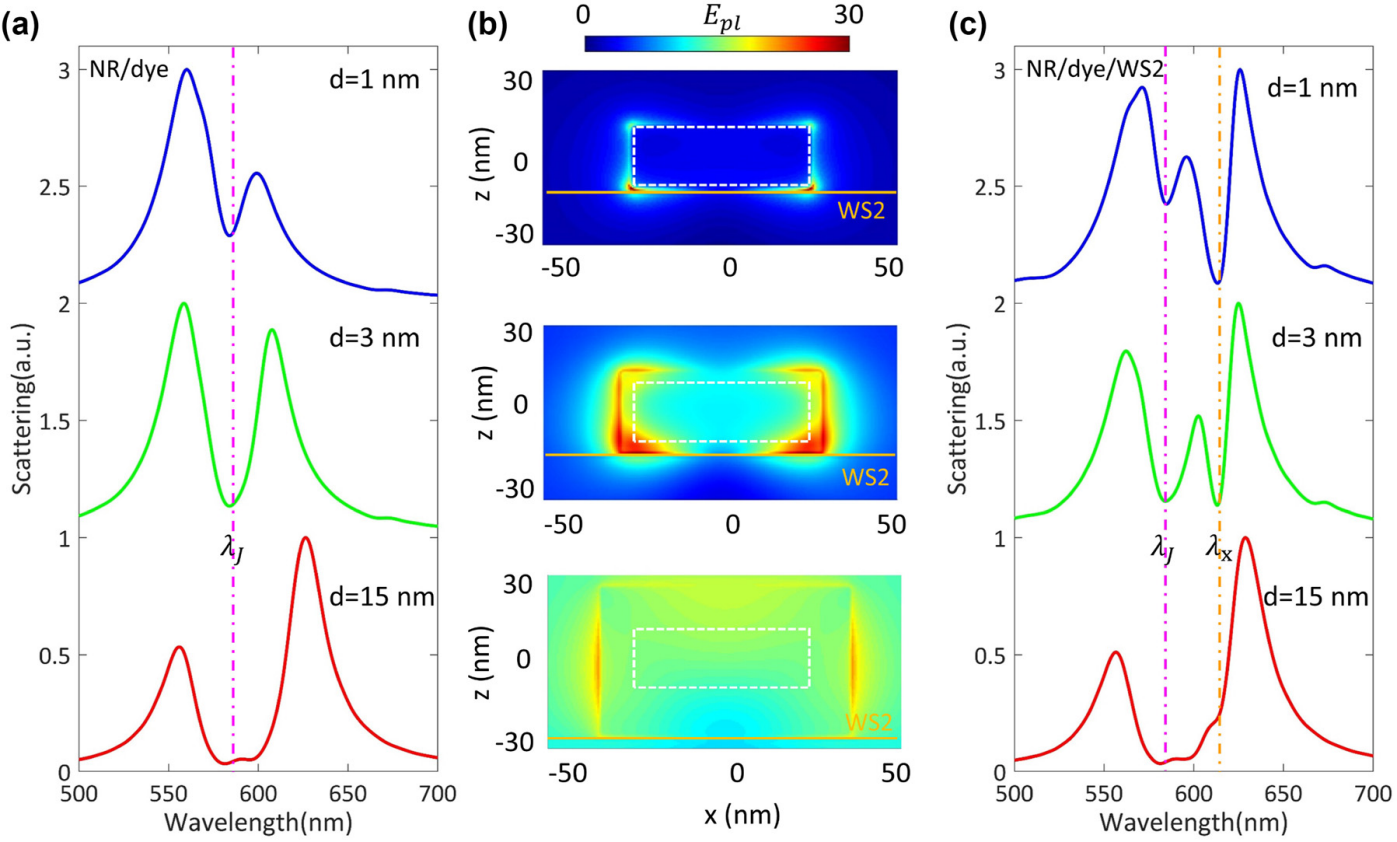
Analysis of mode competition for WS_2_/dye/Au@Ag nanocavity coupled systems. (a) Scattering spectra calculated for Au@Ag nanocavity coated with different J-aggregate thicknesses. (b) The plasmonic cavity E-field distributions calculated at 592/602/612 nm. The white dashed lines indicate the outline of the Au@Ag nanorod. The solid orange lines indicate the position of the WS_2_ monolayer. (c) Corresponding scattering spectra calculated for WS_2_/dye/Au@Ag nanocavity with different J-aggregate thicknesses.

The strong interaction with nanocavity required sufficient electric field enhancement. The mode volume of Au@Ag nanocavity is extremely small (∼240 nm^3^), leading to inevitable mode competition between two excitonic materials within the plasmonic hotspots. Thus, we can trigger and manipulate the biexcitons-plasmon strong coupling by optimizing the interaction distance. For the J-aggregates/Au@Ag nanocavity shown in Figure 3a, the scattering dip appears at ∼592 nm. The Rabi splitting of the nanocavity/dye hybrid system becomes more significant as the J-aggregates layers grow. The corresponding plasmonic cavity E-field distributions in the *xz* planes are shown in Figure 3b. It indicates that more J-aggregates excitons participate in the coupling process as the dye molecular layers become thicker, which leads to larger Rabi splitting in Figure 3a. On the other hand, the dye molecular layers also serve as the spacer layer in the nanocavity/J-aggregates/WS_2_ hybrid system, which significantly impairs the coupling strength between nanocavity and WS_2_. As shown in upper Figure 3c, when the dye molecular layers are rather thin (*d* = 1 nm), the coupling strength between nanocavity and J-aggregates is weak, and the MPB leans towards *λ*_*J*_. In lower Figure 3c, when the dye molecular layers are thick (*d* = 15 nm), the interaction between WS_2_ and nanocavity is almost blocked. The mode competition is well reflected in the MPB resonance. The pink/orange dashed line indicates the location of J-aggregates/WS_2_ excitons resonance. As the J-aggregates layers become thicker, the WS_2_ excitons in the plasmonic hotspots decrease. Interestingly, the MPB resonance is pushed toward WS_2_ resonance as the J-aggregates dominate the hybrid system. On the contrary, the MPB resonance would lean toward J-aggregates resonance when WS_2_ is dominant. We found that MPB would locate at the center of J-aggregates/WS_2_ resonance when the coating thickness of J-aggregates is ∼3 nm. This particular coating thickness ensures both J-aggregates and WS_2_ excitons can actively participate in the strong coupling process, which guides our subsequent experiments.

### 2.4 Biexcitons-plasmon coupling in Au@Agnanocavity/J-aggregates/WS_2_ hybrids

**Figure 4:**
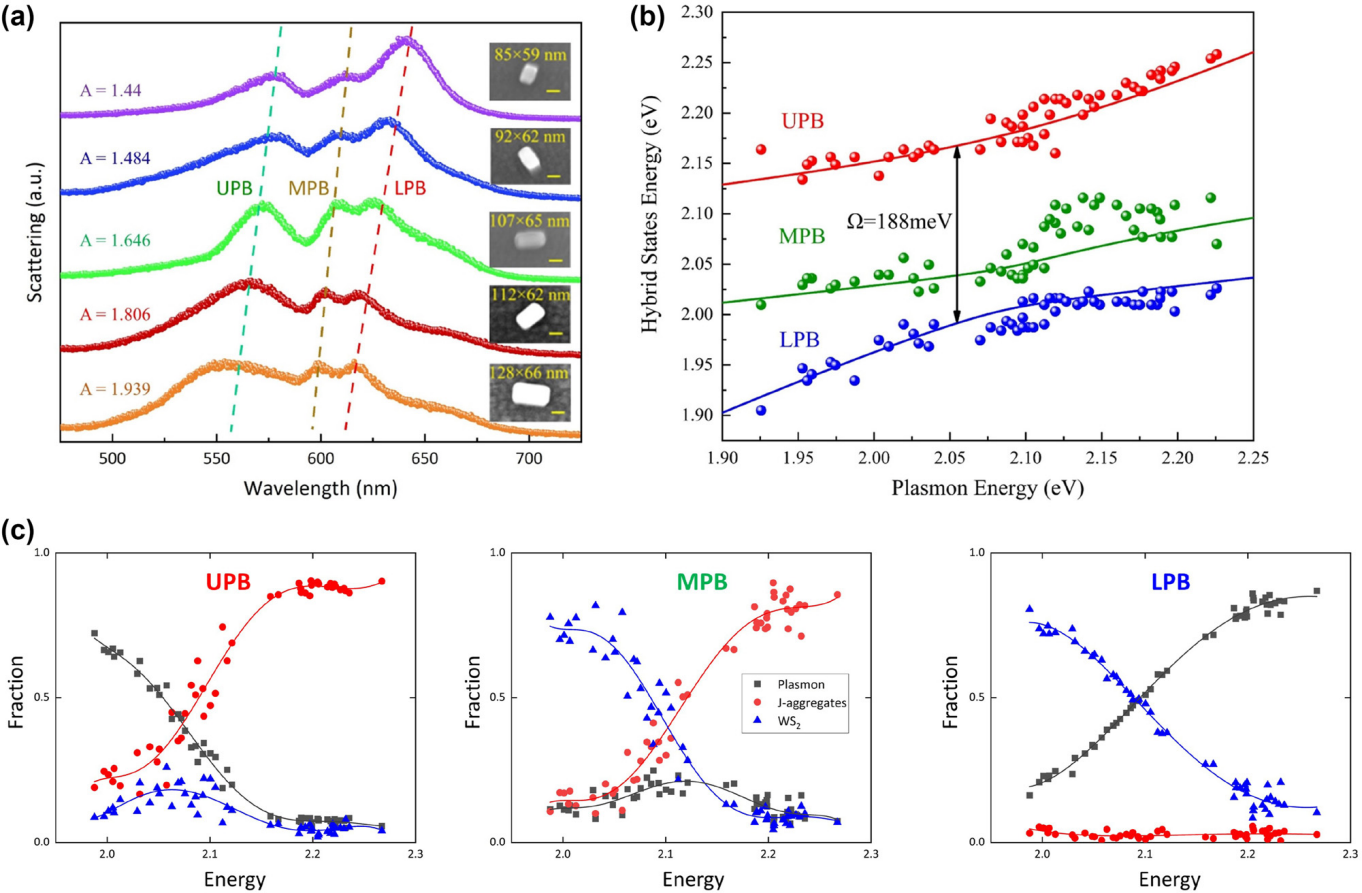
Manipulating the biextions-plasmon coupling with a structure tuning method. (a) Scattering spectra of different plasmon-biexciton strong coupling systems constructed by using Au@Ag nanocavities with different aspect ratios. The SEM and corresponding CCD images of the measured WS_2_/J-aggregates/Au@Ag nanocavities are shown in the insets. (b) Dispersion of the eigenenergies of the three coherent hybrid states. The red/green/blue curves represent the theoretical values for UPB/MPB/LPB, while the colored symbols represent the scattering peaks obtained from the experimental data. (c) Hop-field coefficients for plasmon, J-aggregates exciton, and WS_2_ exciton contributions to UPB, MPB, and LPB states as a function of the plasmon energy.

To construct a plasmon-biexcitons strong coupling nanosystems, we first coat a uniform J-aggregates layer (∼3 nm) on the surface of the Au@Ag nanocavities, then the hybrids were facially integrated with a WS_2_ monolayer. Here the J-aggregates layers were also used as a dielectric spacer layer, significantly impacting the plasmon-WS_2_ coupling strength. We use a correlative dark-field and SEM imaging method to obtain the morphology and spectroscopic information of individual nanocavity/J-aggregates/WS_2_ hybrids. As illustrated in Figure 1a, when both the J-aggregates and WS_2_ monolayer were within the plasmon E-field hotspots, coherent energy transfer between plasmon and biexcitons occurred due to the field enhancement effect of plasmon mode. Consequently, new hybrid polariton states would be produced, as shown in Figure 1b, which is more complicated than traditional two-mode coupled nanosystems. We measured more than 200 dark-field scattering spectra of different nanocavity/J-aggregates/WS_2_ hybrids, where the plasmon resonance was tuned by adjusting the thickness of Ag shells during the synthesis process. Figure 4a shows the scattering spectra of five representative nanocavity/J-aggregates/WS_2_ hybrids, demonstrating the spectral evolution of plasmon-biexcitons coupling in different detuned conditions. Three scattering peaks appear at the flank of the J-aggregates/WS_2_ excitons resonance, corresponding to the three eigenenergies of the plasmon-biexcitons coupling systems. In Figure 4a, as the Ag shell becomes thicker, the aspect ratio of Au@Ag nanocavities increased. The plasmon resonance was tuned to blue-shift, leading to a synchronized blue-shift in all three eigenenergies, while the relative intensity of three scattering peaks would also variate. Meanwhile, the two dips of scattering spectra remain unchanged, corresponding to the exciton resonance of the J-aggregates/WS_2_ monolayer. It provides strong evidence that the new hybrid polariton states result from plasmon/J-aggregates/WS_2_ interaction. For the plasmon-biexcitons coupling in our experiment, the eigenmodes’ energy can be described using the three-coupled oscillator model (TCOM), which is written as [46–49].

(1) $$\left(\begin{array}{ccc} E_{pl}-i\frac{\gamma_{pl}}{2} & g_{j} & g_{x}\\ g_{j} & E_{j}-i\frac{\gamma_{j}}{2} & 0\\ g_{x} & 0 & E_{x}-i\frac{\gamma_{x}}{2} \end{array}\right)\left(\begin{array}{c} \alpha \\ \beta \\ \gamma \end{array}\right)=E\left(\begin{array}{c} \alpha \\ \beta \\ \gamma \end{array}\right)$$

where *E*_*pl*_, *E*_0_, and *E*_*x*_ are the energies for cavity plasmon resonance, J-aggregates exciton resonance and WS_2_ exciton resonance, respectively. Here, *γ*_*pl*_, *γ*_0_, and *γ*_*x*_ represent the corresponding dissipation rates, and *g*_*j*_/*g*_*x*_ reflects the plasmon-J excitons/plasmon-WS_2_ excitons coupling strength. Notably, the J-aggregates/WS_2_ coupled strength is negligible due to the detuning between two excitons. E is the hybrid polariton energies of the three-mode coupling system. *α*, *β*, and *γ* are the Hopfield coefficients. ∣*α*∣^2^, ∣*β*∣^2^, and ∣*γ*∣^2^ indicate the proportion of plasmon, J-aggregates and WS_2_ excitons in the hybrid polariton states, which satisfy ∣*α*∣^2^ + ∣*β*∣^2^ + ∣*γ*∣^2^ = 1. By solving the characteristic equation (1), three unique solutions *E*_*U*_, *E*_*M*_, *E*_*L*_ can be obtained for *E*. In Figure 4b, the theoretical fitting results are shown by three solid curves, which represent the hybrid states of three anti-crossed bands, corresponding to the upper polariton branch (UPB), middle polariton branch (MPB) and lower polariton states (LPB). The resonance energy of plasmon nanocavity *E*_*pl*_ is essential for analyzing anti-crossing behavior in strong coupling systems. However, it can not be directly obtained by experimental measurements because the resonance of plasmonic nanocavities would red-shift after coating with molecule layers. To perform the anti-crossing analysis, we first obtain the corresponding energy for the upper (*E*_*U*_), middle (*E*_*M*_), and lower (*E*_*L*_) polariton branches from dark-field scattering spectra. Then the exciton resonance of J-aggregate (*E*_*j*_) and WS_2_ (*E*_*x*_) is extracted from the absorption measurements. Finally, we obtain the unknown value of *E*_*pl*_ using energy conservation equality: *E*_*pl*_ + *E*_*j*_ + *E*_*x*_ = *E*_*U*_ + *E*_*M*_ + *E*_*L*_. The equality originates from trace invariance of the matrix representation of the Hamiltonian, which was proposed in recent research [26, 50] to analyze three-mode coupling systems.

As shown in Figure 4b, the TCOM fits well with our experimental results, indicating a giant Rabi splitting energy of 188 meV at the center of J-aggregates/WS_2_ excitonic resonances. It is worth noting that the fitting results in the coupling strengths are *g*_*j*_ = 68.7 meV and *g*_*x*_ = 62.1 meV, respectively. Therefore, when the plasmon mode is resonant with J-aggragates/*WS*_2_ excitons, the splittings extracted at zero detuning are Ω_*J*_ = 137 meV(*E*_*pl*_ = *E*_*j*_) and Ω_*X*_ = 124 meV(*E*_*pl*_ = *E*_*x*_). The coupling strengths are slightly decreased compared to the plasmon-single exciton coupling situation but still satisfy the strong coupling criterion of *g*_*j*_ > |*y*_*pl*_ − *y*_*j*_|/4 = 31.25 meV and *g*_*x*_ > |*y*_*pl*_ − *y*_*x*_|/4 = 32.75 meV. Figure 4c shows the calculated Hopfield coefficients, which indicate the proportion of plasmon/J-aggregates/WS_2_ energy in each hybrid polariton state. Specifically, the coupling between J-aggregates and plasmon dominates the UPB, while the coupling between WS_2_ and plasmon dominates the LPB. It results from the detuning between plasmon and J-aggregates/WS_2_ excitons. The coupling strength grows as the detuning decrease. The properties of MPB are more complicated, which contains few proportions of plasmon compared to its excitonic parts. Therefore in Figure 4b MPB shows less disperse than UPB/LPB. Also, since *E*_*J*_ > *E*_*X*_, J-aggregates excitons show more impact in MPB regarding the high energy regime. The strong coupling criterion for three elementary excitations can be expressed as Ω > *α*_1_*κ*_*upb*_ + *α*_2_*κ*_*mpb*_ + *α*_3_*κ*_*lpb*_ [36, 40].

Here, *α*_1_, *α*_2_, and *α*_3_ represent the fractions of the UPB/MPB/LPB in the hybrid polariton states; *κ*_*upb*_, *κ*_*mpb*_, and *κ*_*upb*_ denote the linewidths of the UPB/MPB/LPB. By extracting the zero-detuned data from Figure 4c, the linewidths of each branch can be expressed as follows:

(2) $$\left\{\begin{array}{cc} \kappa_{\mathrm{upb}} & =52.6\;\%\gamma_{pl}+40.7\;\%\gamma_{j}+6.7\;\%\gamma_{x}\\ \kappa_{\mathrm{mpb}} & =22.1\;\%\gamma_{pl}+40.9\;\%\gamma_{j}+37\;\%\gamma_{x}\\ \kappa_{\mathrm{lpb}} & =36.7\;\%\gamma_{pl}+7\;\%\gamma_{j}+56.3\;\%\gamma_{x} \end{array}\right.$$

The following formula can derive the weight coefficients of UPB/MPB/LPB [31]:

(3) $$\left\{\begin{array}{c} \alpha_{1}=\frac{\kappa_{\mathrm{upb}}}{\kappa_{\mathrm{upb}}+\kappa_{\mathrm{mpb}}+\kappa_{\mathrm{lpb}}}\\ \alpha_{2}=\frac{\kappa_{\mathrm{mpb}}}{\kappa_{\mathrm{upb}}+\kappa_{\mathrm{mpb}}+\kappa_{\mathrm{lpb}}}\\ \alpha_{3}=\frac{\kappa_{\mathrm{lpb}}}{\kappa_{\mathrm{upb}}+\kappa_{\mathrm{mpb}}+\kappa_{\mathrm{lpb}}} \end{array}\right.$$

By substituting Equations (2) and (3), we can derive the criterion for the strong coupling of plasmon/J-aggregates/WS_2_ as Ω > *α*_1_ ⋅ *κ*_*upb*_ + *α*_2_ ⋅ *κ*_*mpb*_ + *α*_3_ ⋅ *κ*_*lpb*_ = 74.8 meV. In our case, we measured a relatively large Rabi splitting Ω ≈ 188 meV at zero-detuned point (as shown in Figure 4b), which satisfy the strong coupling criterion. The detuning Ω > *α*_1_ ⋅ *κ*_*upb*_ + *α*_2_ ⋅ *κ*_*mpb*_ + *α*_3_ ⋅ *κ*_*lpb*_.

### 2.5 Adjusting the excitonic resonance of a WS_2_ monolayer by temperature control and fine-tuning the biexcitons-plasmon

**Figure 5:**
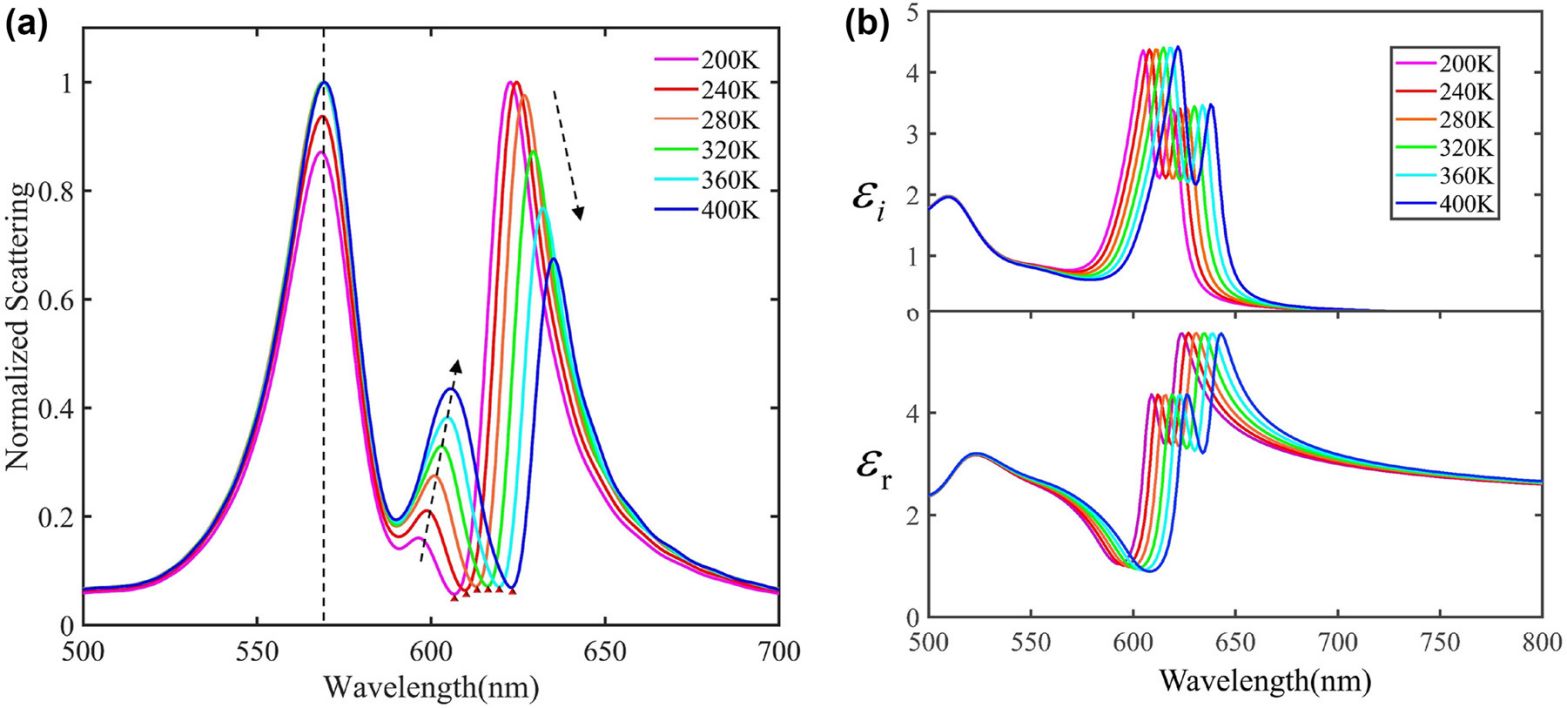
Fine-tuning the biextions-plasmon coupling by temperature control. (a) Normalized scattering spectra calculated for WS_2_/J-aggregates/Au@Ag nanocavities in different temperatures. (b) Complex dielectric constants of a WS_2_ monolayer in different temperature conditions.

Finally, in Figure 5, we demonstrate that the biexcitons-plasmon strong coupling nanosystem can be actively and reversibly manipulated by tuning the environment temperature. WS_2_ monolayer possesses multidimensional adjustable optical properties, for example, its band gap varies with environmental temperature. Figure 5b indicates that the real and imaginary parts of a WS_2_ monolayer would regularly red-shift to a longer wavelength with increasing environmental temperature, which offers us the opportunity to manipulate the biexcitons-plasmon coupling strength. The WS_2_ excitonic energy under different temperatures can be theoretically described by O’Donnell Model [51, 52]. In Figure S3, we calculate the WS_2_ exciton resonance in the 200 K–400 K range using O’Donnell model, which indicates a red-shift from 606 nm to 624 nm. In Figure 5a, we show the scattering spectra of Au@Ag nanocavity/J-aggregates/WS_2_ calculated in different temperatures, which indicates that the middle and lower biexcitons-plasmon hybrid states could be accurately adjusted via temperature control. Here, the aspect ratio of the simulated nanoparticle is set as 1.65 and the plasmon resonance of the nanocavity is 592 nm, which overlay with the J-aggregates’ excitonic energy. The thickness of the coating J-aggregates is set as 3 nm and then facially integrated with monolayer WS_2_. The dashed arrows in Figure 5a shows that the UPB rarely change during the regulation process, while MPB and LPB gradually redshift as the temperature increase. As a result, the Rabi splitting between UPB and MPB increases from 191 meV to 226 meV. Notably, the scattering dips between MPB and LPB (marked with red triangles in Figure 5a) are in accordance with the calculated WS_2_ exciton resonance. The tuning accuracy of MPB/LPB resonance wavelength can reach up to ∼1 nm when the variation of temperature is ∼10 K, which provides a delicate and reversible method to control the biexcitons-plasmon coupling system.

## 3 Conclusions

In summary, we have systematically investigated the spectral properties and manipulating methods of plasmon-biexcitons strong coupling in Au@Ag/J-aggregates/WS_2_ hybrid nanosystems. Three new plexciton branches formed by multimode hybridization are observed from the darkfield scattering spectra, which present a giant exciton-plasmon-exciton energy splitting of ∼188 meV at the center of excitonic resonances. Hopfield coefficient calculations reveal the plasmon-biexcitons have properties that are intermediate between plasmons and J-aggregates/WS_2_ excitons, which enable the possibility to obtain the best features of both metal and semiconductors. Furthermore, we have investigated the mode competition between heterogeneous excitonic materials and proposed the optimal parameters for J-aggregates spacer, which is in the middle range of localized plasmonic hotspots. Accordingly, from the WS_2_ exciton perspective, we have also demonstrated the dynamic control of plasmon-biexcitons via thermal regulation. The temperature-resolved spectra are ascribed to the dielectric constant modification of WS_2_, which significantly affect the coupling strength between plasmon and *X*_*A*_ excitons. Our findings offer a versatile platform to construct and manipulate multiqubit coupling in room-temperature conditions and pave the way for developing diverse plexcitonic devices.

## 4 Methods

### 4.1 Sample preparation

We fabricated the cuboidal Au@Ag nanocavity with sharp edges using a seed-mediated growth method (details are provided in Supporting Information, Section 1). Figure S1 shows the high-resolution TEM image of one typical Au@Ag nanocavities with proper dimensions (diameter ∼48.2 ± 1.4 nm and length ∼78.3 ± 3.1 nm). To boost the plasmon-exciton coupling strength, the curvature radius of the Ag shell at the edgy is tailored to ∼2 nm, causing a dramatic small mode volume down to a few hundred cubic nanometers (see Supporting Information, Figure S2). J-aggregates layers were firmly attached to the surface of Au@Ag nanocavity via electrostatic interaction, which forms a typical plasmon-exciton strong coupling system. The nanocavity/J-aggregates hybrid was then transferred to a bare silicon wafer substrate for subsequent measurements.

### 4.2 Optical measurements

We use a reflection-type dark-field scattering experiment setup to investigate the optical properties of the individual nanocavity. Individual Au@Ag nanocavities were optically characterized using an upright microscope (BX51, Olympus) combined with an imaging spectrograph (IsoPlane 160, Princeton Instrument) and an EMCCD camera (Ultra 888, Andor). The sample surface was illuminated with broadband white light from a Laser-Driven Light Source (EQ-99X, Energetiq), which was focused onto the sample surface using a 100X dark-field condenser (NA = 0.4). In addition, numerical symbols in the silicon wafer were used to locate individual nanocavity in dark-filed and SEM imaging.

## Supplementary Material

Supplementary Material Details
