# Supplementary material for: Fine-tuning biexcitons-plasmon coherent states in a single nanocavity
Source: Nanophotonics. 2023 Jul 25;12(17):3471–80. doi: 10.1515/nanoph-2023-0304 (PMC11501157; doi:10.1515/nanoph-2023-0304)
Supplement: Supplementary file 1 — Supplementary Material Details [file j_nanoph-2023-0304_suppl_001.docx]

**Supplementary Information**

Fine-tuning biexcitons-plasmon coherent states in a single nanocavity

*Kun Liang,*^1^ *Lei Jin,*^2^ *Xuyan Deng,*^2^*Ping Jiang,*^3^ *and Li Yu^*4^*

^1^ School of Electronic Engineering, Beijing University of Posts and Telecommunications, 10 Xitucheng Road Beijing, China

^2^ School of Science, Beijing University of Posts and Telecommunications, 10 Xitucheng Road Beijing, China

^3^ School of Science Microelectronics and Data Science, Anhui University of Technology, Maanshan, China

^4^State Key Laboratory of Information Photonics and Optical Communications, School of Science, Beijing University of Posts and Telecommunications, 10 Xitucheng Road, Beijing, 100876, China

^*^E-mail: [yuliyuli@bupt.edu.cn](mailto:yuliyuli@bupt.edu.cn);

**S1. The synthesis method of Au@Ag nanorods.**

After preparing gold nanorods, we utilized a seed-mediated growth method to gradually coat their surfaces with a uniform layer of silver, forming silver-coated gold nanorods.

**Chemical reagents used in the synthesis process.** benzylhexadecyldimethylammonium chloride (CTAC, 99%), L- ascorbic acid (AA, C_6_H_8_O_6_, >99%), Cetyltrimethylammonium bromide (CTAB≥99%), sodium borohydride (NaBH_4_, 99%), gold (III) chloride trihydrate (HAuCl_4_·3H_2_O, >99%), and sodium chloride (NaCl, 99.5%) were used as received. 5,6-Dichloro-2-[[5,6-dichloro-1-ethyl-3-(4-sulfobutyl)-benzimidazol-2-ylidene]-propenyl]-1-ethyl-3-(4-sulfobutyl)-benzimidazolium hydroxide, inner salt, sodium salt (TDBC) was purchased from Few Chemicals and used as received. Deionized water with a resistivity of 18.25 MΩ.cm was used in all experiments.


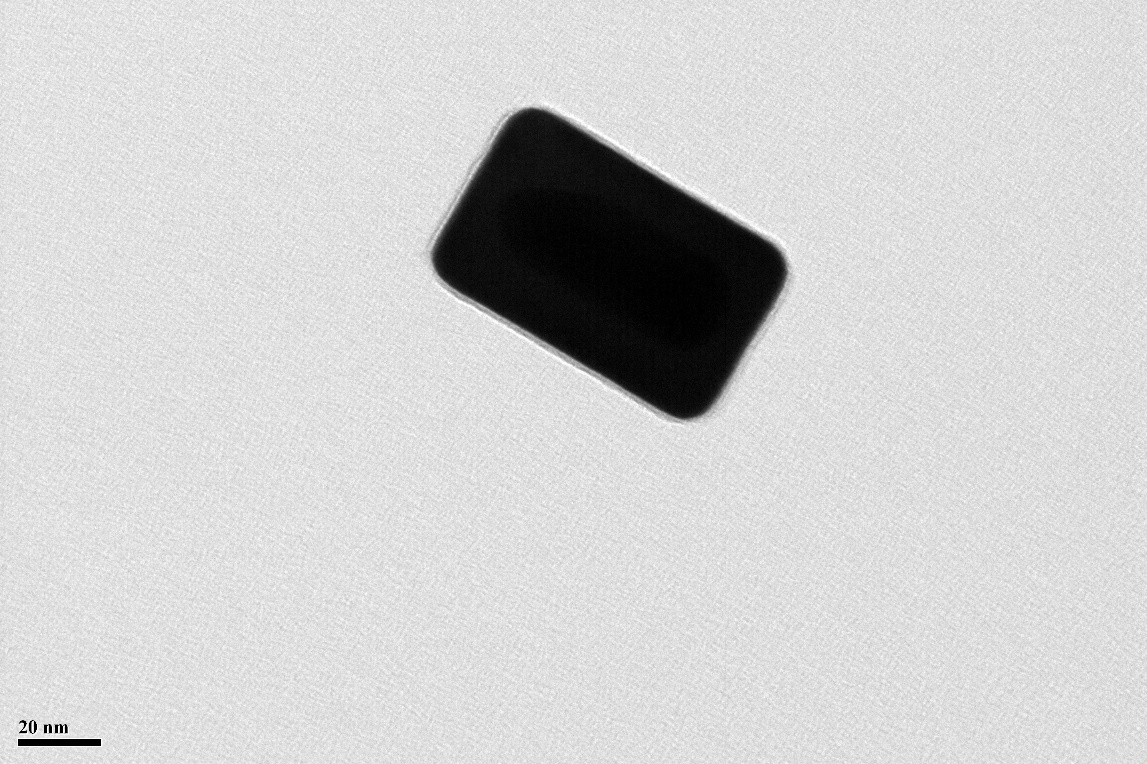


**Figure S1.** The high-resolution TEM image of one typical Au@Ag nanocavity.

**S2. E-field distribution of Au@Ag nanocavity and mode volume calculation**


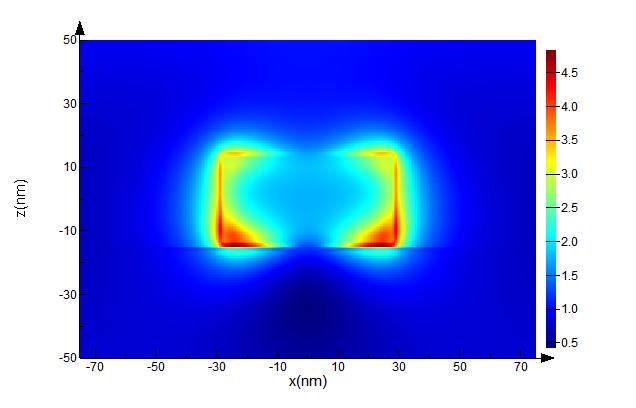


**Figure S2.** Electric field distribution of Au@Ag nanorod, calculated by FDTD simulation. ($\lambda_{cav}=592 nm)$ The nanorod was put on the surface of monolayer $\mathrm{WS}_{2}$. The substrate was set as $\mathrm{SiO}_{2}$ and the background was air.

The mode volume of the Au@Ag nanocavity was calculated using the formula^1,2^:

$$V=\frac{\int\varepsilon\left( r \right)|E\left( r \right)|^{2}dV}{max(\varepsilon(r)|E\left( r \right)|^{2})}$$

$\varepsilon\left( r \right)$ represent the environment dielectric constant at r. Considering the plasmon properties, we change the term $\varepsilon\left( r \right)$ to $Re[\varepsilon]+2\omega Im\left[ \varepsilon\right]/\gamma$ ($\omega$ is the angular frequency of the cavity mode, $\gamma$ is the Drude damping term. For gold material, $\gamma_{Au}\sim0.07.$ For silver material, $\gamma_{Ag}\sim0.02.$)

In a specific calculation, the electric field distributions inside and around the Au and Au@Ag nanorod were calculated at the J-aggregate transition energy (E=2099 meV,λ=592 nm) using the FDTD simulation (Figure S2). Subsequently, the mode volume of Au@Ag nanorod used in our study was calculated to be about 240 ${nm}^{3}$.

**S3. Calculations of the $\mathbf{WS}_{\mathbf{2}}$ exciton resonance**

Here, we calculate the $\mathrm{WS}_{2}$ exciton resonance in 200 K-400 K range using O'Donnell Model^3,4^:

(1)

$E_{g}(0)$=2.07 eV represents the excitonic transition energy at 0 K. S=1.78 represents electron-phonon coupling strength. represents the average phonon energy.


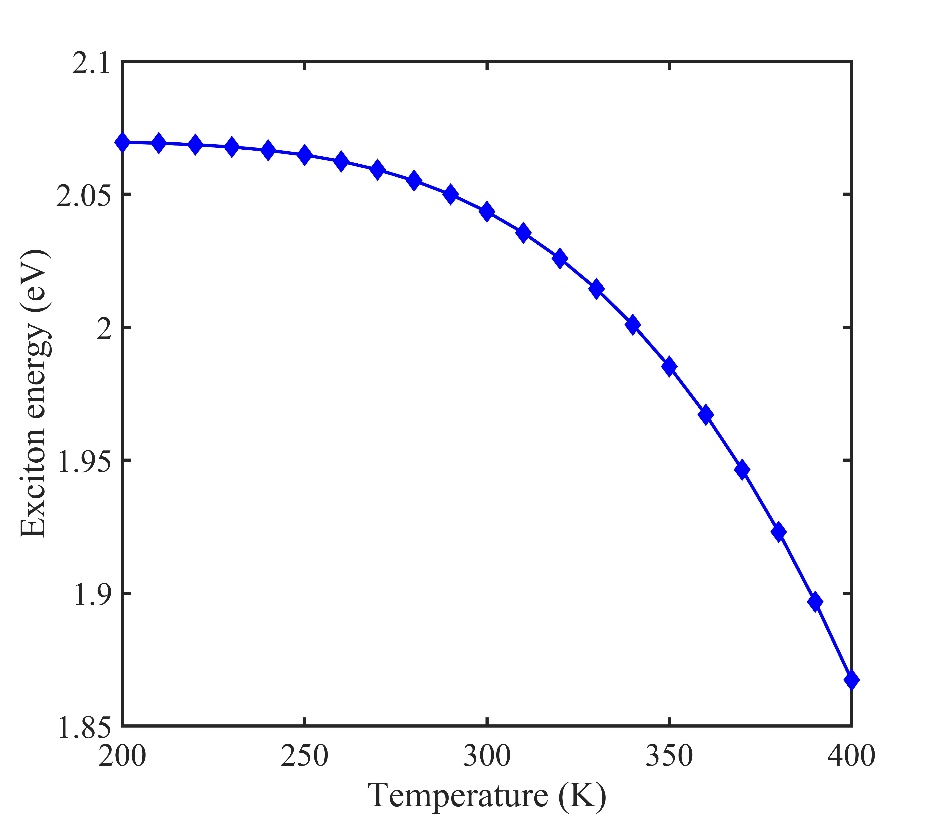


**Figure S3.** The calculated $\mathrm{WS}_{2}$ exciton resonance in 200 K-400 K range.

**S4. Absorption measurement of $\mathbf{WS}_{\mathbf{2}}$ monolayer**

To extract the decay rate of the WS2 excitons, we measured the absorption spectrum of the WS2 monolayer using a typical microscope setup. Firstly, the incident light from a halogen lamp was focused into the sample plane by a 100X magnification objective (NA=0.9). Then, the imaging of the WS2 monolayer was acquired by a color CCD. Finally, the spatial filtering method was used to capture the absorption spectrum of monolayer WS2. As shown in Figure S4, the experimental data agree well with single Lorentz peak fitting results (red line). The WS2 monolayer absorption peak $E_{x\_fit}$= 2.025 eV, and the exciton width is $\gamma_{x\_fit}$= 26 meV.


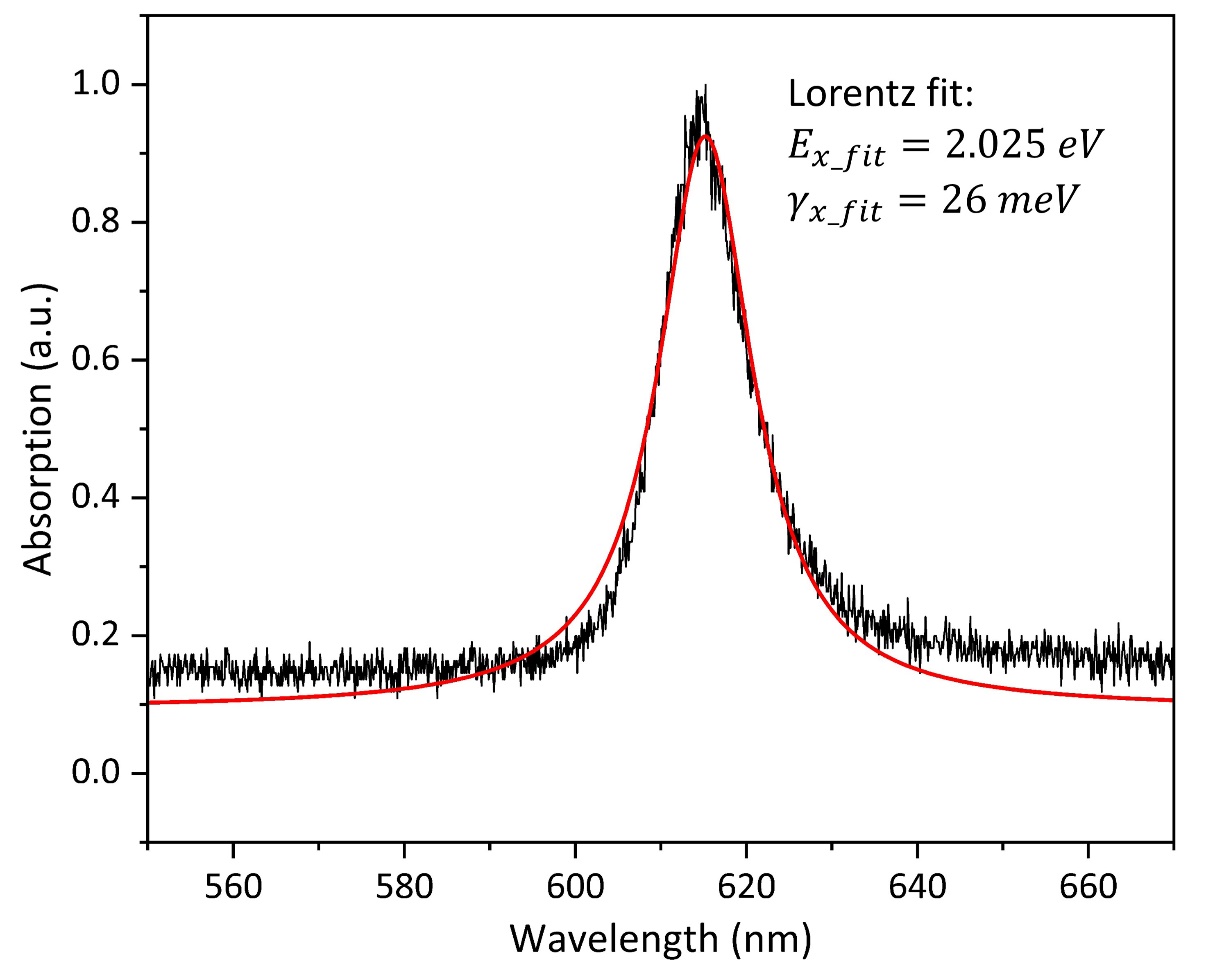


**Figure S4.** The measured absorption spectrum of $\mathrm{WS}_{2}$ monolayer. The red solid line shows the Lorentz fit for the experimental data.

References

1 Zengin, G. *et al.* Realizing Strong Light-Matter Interactions between Single-Nanoparticle Plasmons and Molecular Excitons at Ambient Conditions. *Physical Review Letters* **114**, 157401, doi:10.1103/PhysRevLett.114.157401 (2015).

2 Wen, J. *et al.* Room-Temperature Strong Light–Matter Interaction with Active Control in Single Plasmonic Nanorod Coupled with Two-Dimensional Atomic Crystals. *Nano Letters* **17**, 4689-4697, doi:10.1021/acs.nanolett.7b01344 (2017).

3 O’donnell, K.P., Chen, X. Temperature dependence of semiconductor band gaps. *Applied physics letters* **58**, 2924-2926, doi: 10.1063/1.104723 (1991).

4 Cuadra, J. et al. Observation of tunable charged exciton polaritons in hybrid monolayer WS2– plasmonic nanoantenna system. *Nano Letters* **18**, 1777–1785, doi: 10.1021/acs.nanolett.7b04965 (2018).
